# New distribution and host records for *Hectopsylla pulex* Haller (Siphonaptera, Tungidae) with notes on biology and morphology

**DOI:** 10.3897/zookeys.389.7042

**Published:** 2014-03-14

**Authors:** Michael W. Hastriter, Michael D. Meyer, Richard E. Sherwin, Katharina Dittmar

**Affiliations:** 1 Monte L. Bean Life Science Museum, Brigham Young University, Provo, Utah, U.S.A.; 2 Department of Organismal and Environmental Biology, Christopher Newport University, Newport News, VA 23606; 3 University of Buffalo, SUNY, Department of Biological Sciences, 109 Cooke Hall, Buffalo, NY, 14260

**Keywords:** Arizona, bats, fleas, *Leptonycteris*, neosomy, Phyllostomidae

## Abstract

*Hectopsylla pulex* Haller is documented for the first time from Cochise County, Arizona on the Lesser Long-nosed bat, *Leptonycteris yerbabuenae* Martinez and Villa (Phyllostomidae). This represents the first record of this flea on this Chiropteran Family. The most favorable site of attachment of *H. pulex* appears to be the head, particularly the ears and tragus. The potential interference of echolocation caused by flea attachment at or near the tragus is discussed in addition to host preferences and specialized morphological features. It is demonstrated that expansion of abdominal segments during egg development is a function of mechanical design and not neosomy such as occurs in *Tunga monositus* Barnes and Radovsky, *Neotunga euloidea* Smit and some vermipsyllid fleas.

## Introduction

Members of the flea genus *Hectopsylla* Barrera parasitize birds, small non-volant mammals and bats. A comprehensive review of the genus *Hectopsylla* was provided by Hastriter and Méndez (2000) to include 12 species and a key to the genus *Hectopsylla*. Nine species are parasites of non-volant mammals, two species on avian hosts, and one on bats. Details of distribution and host specificity are outlined in Hastriter and Méndez (2000) for *Hectopsylla* species found on non-volant mammals. Blank et al. (2007) subsequently described a third avian species, *Hectopsylla narium* Kutzschen, from the nostrils and below the tongue of the Burrowing Parrot, *Cyanoliseus patagonus patagonus* (Vieillot) (Psittacidae) from Patagonia. *Hectopsylla psittaci* Frauenfeld, another avian parasite, has a broad distribution in Central and South America, southwestern United States (California) (Nelson et al. 1979, Schwan et al. 1983), and several populations were introduced into aviaries in England and the Netherlands (Hopkins and Rothschild 1953). *Hectopsylla knighti* Traub and Gammons, known only from a single female, was collected from the head of a swift (species unknown) in Michoacán, Mexico. *Hectopsylla pulex* (Haller) is the only hectopsyllid that occurs on bats. Subsequent to Hastriter and Méndez (2000), two additional records of *Hectopsylla pulex* were reported from molossid bats in Brazil (Esbérard 2001, Luz et al. 2009). Three female specimens of *Hectopsylla pulex* are herein reported and represent new host and distributional records.

## Materials and methods

As part of a long-term ecological study of insectivorous bats in the southwestern United States between 23 June and 4 September 2013, 23 Lesser Long nosed bats, *Leptonycteris yerbabuenae* Martinez and Villa (Phyllostomidae), were captured using a mist net placed adjacent to a hummingbird feeder. Bats were weighed, measured, and examined and fleas were removed with forceps and preserved in 70% ethanol pending processing. A total of three female fleas were collected from the ears of a young adult male *Leptonycteris yerbabuenae*. One flea is deposited in the Brigham Young University DNA flea voucher collection and the other two in the collection of Christopher Newport University. Images were prepared using an Olympus BX61 Compound Microscope, Olympus CC12 digital camera accompanied with an Olympus Microsuite™ B3SV program and Adobe Photoshop, CS4.

## Results and discussion

**Figure 1.**
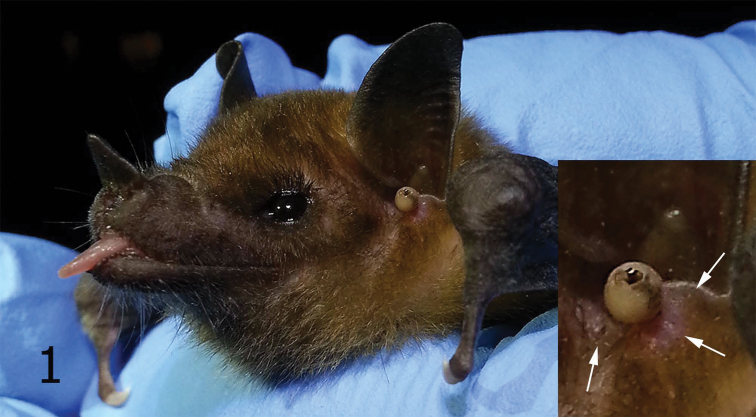
Young male *Leptonycteris yerbabuenae* with *Hectopsylla pulex* attached near tragus. Insert is enlargement of attached *Hectopsylla pulex* (arrows indicate previous flea attachment sites).

A single attached replete female flea was observed anterior to the tragus of *Leptonycteris yerbabuenae* captured at Paradise, Cochise County, Arizona on 23 June 2013. This specimen was not collected but a cellular phone photo was taken to document what probably represents a replete female *Hectopsylla pulex*. Although tentative, this identification is supported by the facts that *Hectopsylla pulex* is: 1) the only representative of the genus that occurs on bats 2) it is the only member of the genus previously reported in the United States, 3) it was present in the same locality, and 4) that it was present in simultaneous collections of *Hectopsylla pulex* on the same host species (*Leptonycteris yerbabuenae*). On 27 July 2013, three additional female *Hectopsylla pulex* specimens were observed and removed from the anterior base of the tragus of *Leptonycteris yerbabuenae* captured in White Tail Canyon, Chiracahua Range, Cochise County, Arizona. A photograph was taken of the one flea prior to its removal from the left ear (Fig. 1), while the other two specimens attached at the same site on the right ear were collected but not photographed. No additional fleas were noted on the other 21 *Leptonycteris yerbabuenae* specimens examined.

**Figures 2–3.**
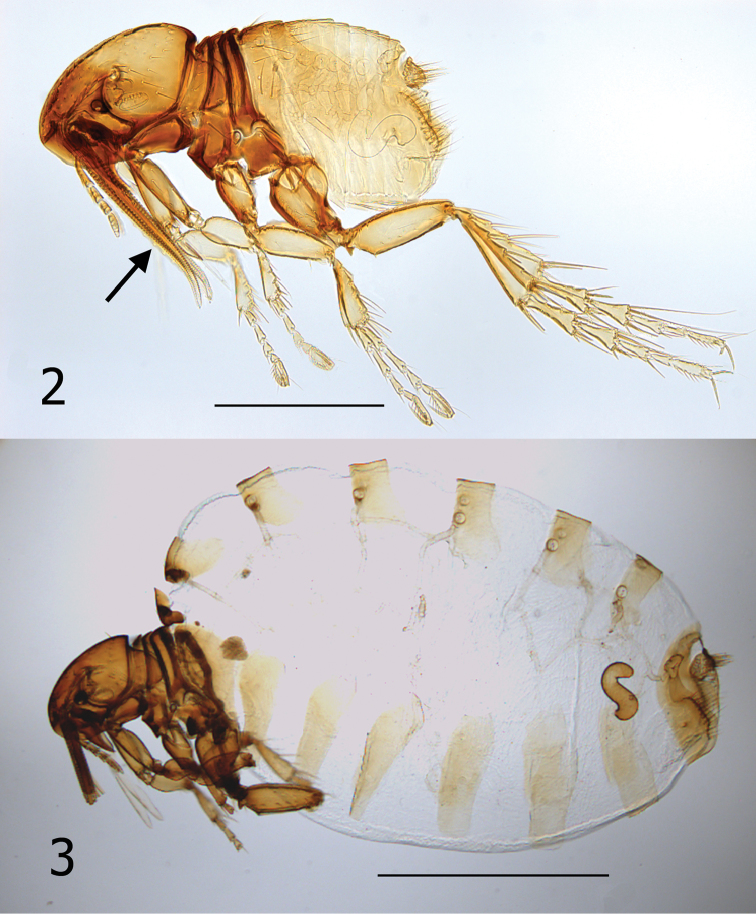
*Hectopsylla pulex*. **2** Overview of an unfed female, Pacora, Panama collected from bat guano. Arrow indicates lacinae **3** Overview of a replete gravid female, host: *Leptonycteris yerbabuenae*, White Tail Canyon, Chiracahua Range, Cochise County, Arizona.

Modest swelling of the skin of the bat adjacent to the base of the attached flea (Fig. 1, arrows) possibly indicates residual scarring from previously attached stick-tight fleas. These fleas attach and feed for extended periods. Little is known about their biology; however, other stick-tight fleas, e.g., *Echidnophaga gallinacea* (Westwood), *Juxtapulex echidnophagoides* Wagner and *Tunga penetrans* (Linnaeus), attach to their host with similar lacinae that are serrated along their margin to enable attachment (Fig. 2, arrow). This group of fleas represents two types of stick-tight fleas: 1) those that attach, feed, and drop off, and 2) those that attach, feed to repletion, become gravid, and ultimately die *in situ*. The first type attaches, retain their legs, and eventually detach and fall off of the host. These may feed multiple times. The second type that includes *Tunga penetrans* and *Hectopsylla pulex* remain attached and autosever their legs as often noted by black scarring of the apices of each severed leg. Severing usually occurs at the apices of the coxae or femora.

**Figures 4–5.**
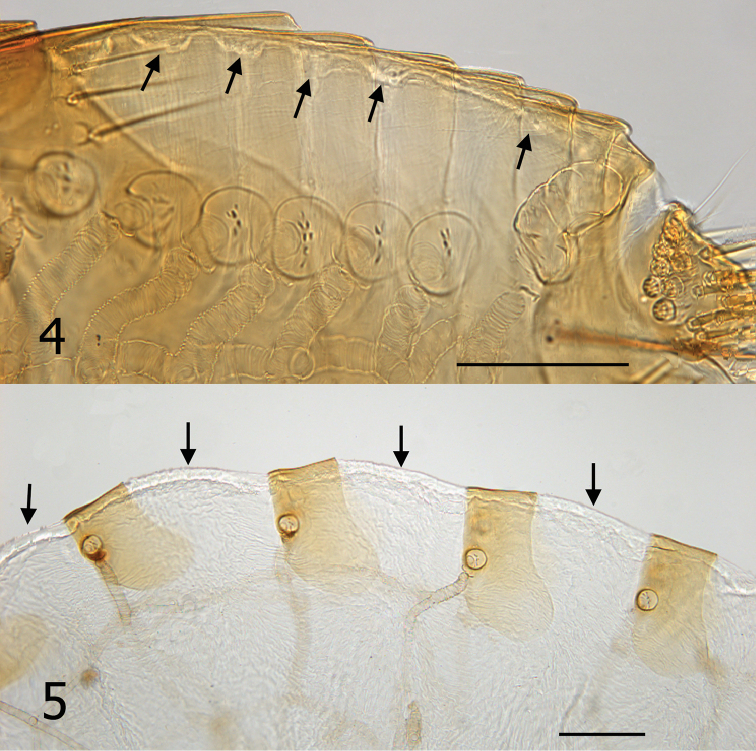
Tergites of *Hectopsylla pulex*. **4** Unfed non-gravid female from Pacora, Panama; arrows indicate membrane prior to expansion **5** Replete gravid female, arrows indicate expanded membrane to accommodate expansion from feeding and egg development.

It is unknown whether males of *Hectopsylla pulex* copulate with females on or off the host. Males of *Hectopsylla pulex* have never been found attached to a host and males have been collected only from bat guano of molossid bats (Traub and Gammons 1950, Méndez 1977). This would suggest that they copulate prior to the female acquiring a host. Following attachment, females become replete with eggs and the abdomen expands many times its original size (compare Figs 2 and 3). Overall length of abdomen of unfed female (Fig. 2) is 620 µm compared to 2400 µm for the fed replete female (Fig. 3). Comparison of an unfed female from Panama and our fed gravid specimen elucidates that the great expansion is not a feature of neosomic development that is documented in *Tunga monositus* Barnes and Radovsky, *Neotunga euloidea* Smit and some vermipsyllid fleas, but rather a pre-established expansible design. This can clearly be seen in Figs 4 and 5. Arrows, Fig. 4, indicate a hyaline membrane folded under the tergites. In addition, the tergites are compressed under one another in a “shingle” fashion (Fig. 4). The fed and expanded female (Fig. 5) illustrates the fully expanded tergites and the membrane that stretches between the posterior margin of the preceding sclerite and the next sclerite (arrows). After eggs develop, eggs are expelled out onto the host and fall onto the substrate. Only eggs that fall into the guano deposits near host colonies (roosting structures) have any chance of completing the life cycle (egg, larva, pupa and imago). Newly emerged imagines then acquire a new host near the guano deposits.

*Hectopsylla pulex* was reported by Hastriter and Méndez (2000) on 13 bat species represented by three families and eight genera: Molossidae (4 genera), Vespertilionidae (3 genera), and Noctilionidae (1 genus). Although a relatively common flea, our finding on *Leptonycteris yerbabuenae* represents the first report on this genus and the family Phyllostomidae. This flea has been documented only once in the United States by Augustson and Ryan (1948) in Medina and Uvalde Counties, Texas on the Mexican Free-tailed bat *Tadarida brasiliensis* (I. Geoffroy). The occurrence of *Hectopsylla pulex* in Cochise County, Arizona is a new State record and also represents the most northern and western limit for the species.

According to Luz et al. (2009), the most common attachment sites for *Hectopsylla pulex* parasitizing *Molossus rufus* É. Geoffroy and *Molossus molossus* (Pallas) were the ear and tragus. The ear was the site of attachment for 26 of 50 *Molossus rufus* examined, while 15 were attached to the tragus. Of the 19 specimens of *Molossus molossus* examined, 12 were attached to the ear and five to the tragus.

Only 11 of the total 69 fleas taken from these two bat species had fleas attached on areas other than the ear and tragus. Esbérard (2001) also reported the head as the most common site of attachment on *Molossus molossus*. Chen and Moss (2005) determined that the pinna and tragus of *Eptesicus fuscus* Palisot de Beauvois, play important roles in filtering returning echos. They found that any manipulation of the tragus adversely affects the bat’s ability to acquire prey and evade obstacles. The large size of the replete *Hectopsylla pulex* female is nearly as large as the tragus of *Leptonycteris verbabuenae* (see Fig. 1). The potential adverse effect of this on the bats echolocation ability may be significant. Additional collecting of this flea from *Leptonycteris yerbabuenae* is needed to evaluate if the attachment site immediately at the anterior base of the tragus is a common phenomenon and a potential impairment of the bat’s echolocation. *Leptonycteris yerbabuenae* are nectivorous and feed primarily on species of Agave. Hence, their echolocation ability may be less important than that of insectivorous bat species that require acrobatic agility to capture insects in flight. On the contrary, molossid bats are insectivorous and may be at a disadvantage with fleas attached near the tragus or other parts of the ear pinna. This might be a fascinating area for additional research.

Comparison of *Hectopsylla pulex* from Panama (Tipton and Méndez 1966) and Arizona demonstrate minor differences. The head of Arizona specimens is only slightly convex at posterior occipital area, whereas the occipital area of Panamanian specimens is more convex. There are also minor differences in the metepimeron, tergum I, and tergum II. Arizona specimens have a heavily sclerotized plate on the anterior portion of the metepimeron bearing six setae and the dorsal portion of the bases of terga I and II are more heavily sclerotized. The overall dimensions of Arizona specimens are slightly larger, resulting in comparably larger measurements of spiracle diameters, head length, and length and width of the vermiform spermatheca.
